# Long Non-coding RNAs in the Regulation of the Immune Response and Trained Immunity

**DOI:** 10.3389/fgene.2020.00718

**Published:** 2020-07-24

**Authors:** Manuel Flores-Concha, Ángel A. Oñate

**Affiliations:** Laboratory of Molecular Immunology, Department of Microbiology, Faculty of Biological Sciences, Universidad de Concepción, Concepción, Chile

**Keywords:** immune response, long non-coding RNAs, trained immunity, gene regulation, epigenetic

## Background

The lncRNAs are a group of transcripts with low or no coding potential, they are defined as transcripts that exceed 200 nucleotides in length, a cut-off point that distinguishes them from smaller non-coding RNAs (ncRNAs) such as transfer RNAs (tRNAs) or microRNAs (miRNAs) (Mathy and Chen, 2017). Based on its relative position with respect to the genetic loci, the lncRNA can be classified as long intergenic non-coding RNAs (lincRNAs), intronic lncRNAs, antisense lncRNAs and enhancer RNAs (eRNAs) (Mattick and Rinn, 2015; Chen et al., 2017). Currently, the GENCODE human genome database has 17,960 documented lncRNA genes, a figure that is not so far from 19,959 protein-coding genes (GENCODEv34). LncRNAs participate significantly in various biological processes, including; transcription (Espinoza et al., 2004; Willingham, 2005; Anderson et al., 2016), splicing (De Troyer et al., 2020), protein localization (Leucci et al., 2016; Munschauer et al., 2018), cell cycle, proliferation and apoptosis (Yang et al., 2018; Rossi et al., 2019; Shen et al., 2020). Transcriptomic analysis in different tissues has shown that the expression profiles of lncRNAs vary according to the type of cell, the stage of development and the physiological conditions to which cells are exposed (Cabili et al., 2011; Mattick, 2011). From the above, it is reasonable to suppose that the modification of the transcriptional profile or the timing of expression of lncRNAs, can contribute to the development of various pathologies (Li et al., 2017; Katsel et al., 2019; Wu et al., 2020; Yao et al., 2020). Although, the study of the roles they play during the immune response is recent and limited to a few of them (Elling et al., 2016; Lewandowski et al., 2019), evidence of their interaction with transcription factors such as NF-κB (Rapicavoli et al., 2013) and STAT3 (Wang et al., 2014), histone-modifying enzymes such as EZH2 (Ranzani et al., 2015, p. 4) and HDAC1 (Castellanos-Rubio et al., 2016) and chromatin remodeling complexes such as SWI/SNF (Hu et al., 2016), demonstrate that lncRNAs actively participate in the coordination of gene expression required by immune system cells and events like the establishment of trained immunity (Table 1).

**Table 1:** Functions of some LncRNAs related to the modulation of gene expression associated with the immune response.

**LncRNA**	**Interaction**	**Function**	**References**
LincRNA-Cox2	hnRNP A/B, hnRNP A2/B1, SWI/SNF, Mi2/NuRD, NF-κB	Modulates chromatin remodeling and transactivation of the late-primary inflammatory response genes.	Carpenter et al., 2013; Hu et al., 2016; Tong et al., 2016; Xue et al., 2019
PACER	NF-κB (p50)	Promote ptgs2 transcription.	Krawczyk and Emerson, 2014
LincRNA-EPS	hnRNPL	Suppresses the expression of immunity-related genes such as Il6, Il1a and Ccl5.	Atianand et al., 2016; Mumbach et al., 2019
Lnc-13	hnRNPD y HDAC1	Suppress gene expression	Castellanos-Rubio et al., 2016
Lethe	NF-κB (p65)	Decreases the translocation of NF-κB and reduces the occupancy of the transcription factor on gene promoters regulated by NF-κB.	Rapicavoli et al., 2013
Lnc-DC	STAT3	Prevents STAT3 dephosphorylation.	Wang et al., 2014
UMLILO	WDR5-MLL1	Promote the epigenetic priming of CXCL8, CXCL1, CXCL2 and CXCL3. Involved in trained immunity.	Fanucchi et al., 2019
LincRNA-MAF-4	LSD1, EZH2	promotes epigenetic silencing of MAF to control differentiation.	Ranzani et al., 2015
LincR-*Ccr*2-5'AS	Not known	Reduces the expression of Ccr1, Ccr3, Ccr2 and Ccr5 genes	Hu et al., 2013
NRON	NFAT, Importin-beta1, LRRK2	Reduces nuclear NFAT, decreasing IL-2 production	Willingham, 2005; Liu et al., 2011

## Involvement of LncRNAs in Immune Response

**Figure 1:**
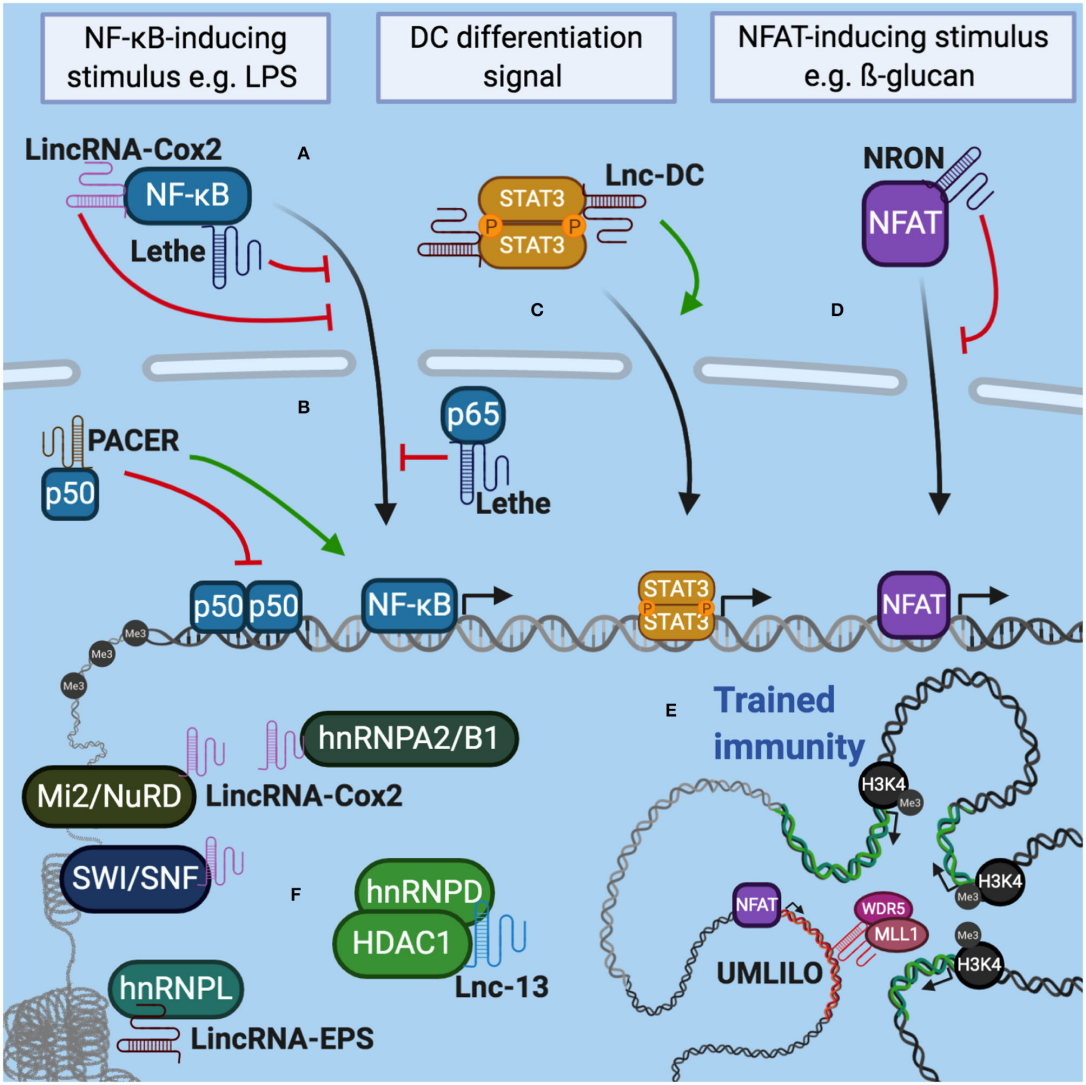
LncRNAs interact with molecular complexes that regulate the expression of genes associated with the immune response. **(A)** Lethe and LincRNA-Cox2 can interact with NF-κB in the cytoplasm and control its translocation to the nucleus. **(B)** In the nucleus, Lethe modulates inflammation by sequestering p65 from NF-κB. PACER interacts with p50 decreasing the formation of homodimers and favoring the heterodimerization of NF-κB. **(C)** In dendritic cells, lnc-DC prevents STAT3 dephosphorylation, promoting its translocation to the nucleus and the expression of genes related to cell differentiation and activation status. **(D)** In lymphocytes NRON interacts with NFAT to prevent its transit to the nucleus, decreasing the expression of IL-2. **(E)** The co-regulation of the expression of distant immune genes grouped in the same TAD is mediated by IPL (for example, UMLILO). IPL expression recruits WDR5/MLL histone methyltransferase complex, promoting the epigenetic priming of immune genes contained in TAD. **(F)** LncRNAs can also interact with hnRNP complexes, chromatin remodeling complexes and histone deacetylases to modulate genome accessibility and control gene expression related to the immune response.

One of the first and most notable examples of lncRNAs being involved in the regulation of the immune response is LincRNA-Cox2. In murine phagocytes stimulated by LPS, lincRNA-Cox2 significantly increases its expression in conjunction with the Cox-2 coding gene (ptgs2) (Carpenter et al., 2013). Its nuclear interaction with various protein complexes like heterogeneous nuclear ribonucleoproteins (hnRNPs) A/B and A2/B1 (Carpenter et al., 2013) or non-fermentable switch/sucrose (SWI/SNF) (Hu et al., 2016), allows it to modulate the expression of different types of immune genes. In epithelial cells, lincRNA-Cox2 can block the expression of Il12b through the recruitment of Mi-2/nucleosome remodeling and deacetylase (Mi2/NuRD) complex to the promoter region of this gene (Tong et al., 2016). LincRNA-Cox2 can exercise its regulatory functions beyond the boundaries of the nucleus, interacting directly with NF-κB, promoting its translocation and recruitment to promoter regions of the Nlrp3 and Asc genes, favoring the activation of inflammasome (Figures 1A,F) (Xue et al., 2019). In human macrophages, another ptgs2-related lncRNA is PACER (P50-associated extragenic RNA COX-2). Its function is to hijack NF-κB1 subunits from the ptgs2 promoter, whose dimerization exerts a repressive effect. In this way, PACER facilitates the formation of NF-κB p65/p50 dimers, promoting the recruitment of p300 histone acetyltransferase to increase the accessibility of polymerase II pre-initiation complexes and guarantee the transcription of ptgs2 (Krawczyk and Emerson, 2014).

In murine resting myeloid cells, the transcription of several immunity-related genes (IRGs) is repressed by the expression of lincRNA-EPS (Atianand et al., 2016). In bone-marrow-derived macrophage (BMDM), both stimulation with LPS and infection with Listeria monocytogenes or Sendai virus, decrease the expression of this lncRNA. LincRNA-EPS is associated with hnRNPL to control nucleosomal positioning and chromatin accessibility. On the other hand, BMDMs obtained from lincRNA-EPS global knockout (KO) mice stimulated with LPS, have high levels of histone H3 trimethylation at lysine 4 (H3K4me3) in IRG promoters such as Il6, Il1a and Ccl5 typically associated with less dense nucleosome structures and active gene promoters assets (Wang et al., 2008). Consistently, lincRNA-EPS deficient mice show higher expression of IRGs and greater susceptibility to septic shock when compared to wild type mice (Atianand et al., 2016; Mumbach et al., 2019). Similarly, in non-stimulated human macrophages, lnc-13 interacts with hnRNPD and HDAC1 to suppress the transcription of immune genes (Figure 1F). An interesting fact is that several patients with celiac disease have a SNP in lnc-13, which prevents their interaction with HDAC1, increasing the expression of immune genes typical of the pathogenesis of this disease (Castellanos-Rubio et al., 2016).

As already mentioned, the interaction with transcription factors is also one of the strategies used by lncRNAs to exercise their modulating role. Lethe, a lncRNA expressed in response to TNF-α, exerts inflammatory regulatory functions through its interaction with the p65 subunit of the NF-κB transcription factor. Lethe transcription decreases the translocation of NF-κB and reduces the occupancy of the transcription factor in gene promoters regulated by NF-κB, within which the Lethe promoter is included, self-regulating its expression (Figures 1A,B) (Rapicavoli et al., 2013). On the other hand, Lnc-DC regulates the differentiation and activation of dendritic cells in humans through its interaction with the transcription factor STAT3. This lncRNA is located in the cytoplasm to bind STAT3 and prevent its interaction with tyrosine phosphatase SHP1. This way, Lnc-DC keeps STAT3 in its active state, promoting the transcription of the program of genes that depend on it (Figure 1C; Wang et al., 2014).

A little more unknown but not less important is the role that lncRNAs play in the adaptive immune response. In humans, the differentiation of naive T lymphocytes to Th1 cells is controlled by lincRNA-MAF-4, which promotes the epigenetic silencing of MAF, a Th2 cell transcription factor. This lncRNA interacts with the LSD1 and EZH2 transcriptional repressors to place the H3K27me3 mark in the MAF promoter and suppresses its expression. The transcription of lincRNA-MAF-4 is exclusive to Th1 lymphocytes and it is not expressed in naive T cells or any other lymphoid lines (Ranzani et al., 2015). On the other hand, Th2 lymphocytes specifically express a lncRNA, dependent on the Th2 “master regulator” GATA3, called LincR-*Ccr*2-5′AS. Using a cell transfer model, it was shown that the decrease of LincR-*Ccr*2-5'AS using shRNA reduced the expression of Ccr1, Ccr3, Ccr2, and Ccr5, as well as the migration of Th2 effector cells to the lung of recipient mice (Hu et al., 2013). In memory CD4 + T cells, the production of IL-2 in response to TCR signaling depends on the activation of the nuclear factor of activated T cells (NFAT) (Dienz et al., 2007). This process can be regulated by NRON, a lncRNA that acts as a repressor, reducing NFAT levels in the nucleus. For this, NRON forms a complex with importin-beta1 and LRRK2, controlling the nucleocytoplasmic traffic of the NFAT (Figure 1D; Willingham, 2005; Liu et al., 2011).

## LncRNAs in Trained Immunity

Traditionally, immunological memory has been a characteristic reserved only for adaptive immunity cells. However, several studies have shown that innate immune cells, are capable of generating a type of immune memory known as trained immunity or innate immune memory, in response to prolonged exposure of microbial components, thus being able to remember transcriptional responses and even inherit these memories to their progeny (Kleinnijenhuis et al., 2014; Kaufmann et al., 2018; Hole et al., 2019). For instance, macrophages derived from trained hematopoietic stem cells (HSCs) are epigenetically reprogrammed and as a result can strongly express immune genes, increasing their ability to resolve an infection (Kaufmann et al., 2018; Mitroulis et al., 2018). The development of this type of immune memory is accompanied by a stable accumulation of epigenetic marks on promoters of multiple immune genes allowing the characteristic priming of innate memory (Hole et al., 2019). Although, it is not very clear how these marks are specifically accumulated on immune genes of trained cells, a recent study shows an active participation of a group of lncRNAs, called Immune-gene priming lncRNAs (IPLs), in the trained immunity (Fanucchi et al., 2019).

On the other hand, several immune genes that are distant from each other (from a one-dimensional perspective), but functionally related, can be spatially grouped into three-dimensional chromatin microdomains called TAD (topologically associating domains), allowing their proximity and co-regulation. Interestingly, many TADs integrate regions that encode lncRNAs, which are actively transcribed along with co-regulated genes (Fanucchi and Mhlanga, 2019; Fanucchi et al., 2019). In this context, IPLs are described as priming mediators of immune genes located in the same TAD that contains them. These lncRNAs are transcribed within these microdomains in order to direct histone modifying enzyme complexes to the set of co-regulated gene promoters, located in the same TAD, inducing their priming by epigenetic labeling. A good example is the upstream master lncRNA of the inflammatory chemokine locus (UMLILO). This IPL contacts the TAD of ELR+ CXCL chemokine genes (which contains the genes CXCL8, CXCL1, CXCL2 and CXCL3), recruiting the WDR5–MLL1 complex to direct it toward the promoters of the genes present in the TAD and catalyze the epigenetic priming, placing the H3K4me3 brand (Figure 1E). In mice, the TAD that groups these chemokines lacks an IPL, therefore the expression of these chemokines cannot be trained. Surprisingly, the insertion of UMLILO in the TAD of murine macrophage chemokines resulted in the training of Cxcl genes, providing strong evidence that lncRNA-mediated regulation is essential for the establishment of trained immunity (Fanucchi et al., 2019).

## Concluding Remarks

The study and identification of lncRNAs as active elements of gene regulation during the immune response adds a new level of complexity to its analysis. Its ability to dialogue with several proteins, allow them to control aspects ranging protein traffic to remodeling of the chromatin architecture. Despite the existence of several reviews that include the role of lncRNAs in immunity, we consider it important to highlight the role that they play in establishing trained immunity, a feature of the immune system that has even been recently considered as a tool for reducing susceptibility and severity of SARS-CoV2 infection (Netea et al., 2020). Until now, the genetic approaches used by (Fanucchi et al., 2019) suggests that the “memory implantation,” require the local expression of the immune-gene priming lncRNA (IPL).

## Future Perspective

If modulation on epigenetic regulators can affect a large number of genes and therefore exhibit unwanted effects, the use of lncRNAs could provide the specificity that is required.

Understanding the factors that dictate the folding state of a given lncRNA, as well as the identification of structural motifs involved in the formation of multicomponent-complexes, may contribute to the design of new therapies. Based on the above, we infer that future advances in the study of lncRNAs could focus on their active participation in the establishment of trained immunity, which could be the beginning for the development of a new generation of immunomodulators, which point to the priming of innate immunity cells, making it more efficient against an immune reaction, as well as helping reverse immunotolerance states. Supplementing the knowledge about lncRNAs with the use of local expression tools (Xu et al., 2019), could bring us closer to obtaining the precision we are looking for.

## Author Contributions

All authors listed have made a substantial, direct and intellectual contribution to the work, and approved it for publication.

## Conflict of Interest

The authors declare that the research was conducted in the absence of any commercial or financial relationships that could be construed as a potential conflict of interest.
